# Loratadine-Loaded Thermoresponsive Hydrogel: Characterization and Ex-vivo Rabbit Cornea Permeability Studies

**Published:** 2018

**Authors:** Behzad Sharif Makhmalzadeh, Anayatollah Salimi, Aida Niroomand

**Affiliations:** a *Nanotechnology* *Research* *Center, Ahvaz* *Jundishapur* *University* *of* *Medical* *Sciences,* *Ahvaz, Iran.*; b *Department* *of* *Pharmaceutics, School* *of* *Pharmacy, Ahvaz* *Jundishapur* *University* *of* *Medical* *Sciences, Ahvaz, Iran.*

**Keywords:** Hydrogel, Thermo-responsible, Ocular drug delivery, Loratadine, Corneal permeability

## Abstract

Poor bioavailability of ophthalmic drops is mainly due to drainage through the nasal-lacrimal duct and a very low permeability through corneal epithelium. The aim of our study was to prepare and characterize an ocular hydrogel of loratadine, as an example of a lipophilic drug, to increase drug concentration and residence time at the site of action in the eye. In this study, a 2^3 ^full factorial design was employed to design and compare the properties of eight different loratadine containing hydrogel formulations. Results showed a significant correlation between the swelling and porosity ratios of the hydrogels and the Pluronic percentage and Pluronic/carbomer ratio in the formulations. Moreover, the release profiles showed fast and sustained release of all the formulations. Evaluation of hydrogels structure by the FT-IR technique indicated that Pluronic interacts with hydroxyl and carboxylic groups in carbomer, which is the main reason of the hydrogel network formation and interacts with loratadine.The permeation of loratadine through rabbit cornea showed that drug permeation percentages for the F2 and F7 formulations were 15 and 70 folds more than that of the control.

## Introduction

The eye is an organ that responses very quickly and severely to the existence of exogenous substances. The unique anatomy*, *physiology, and biochemistry of the eye protect the organ from various exogenous and endogenous insults. Due to the many anatomical and physiological barriers existing in the eye, formulator’s job is to transport drug across these barriers without causing the permanent tissue damage.

There are several routes of administration for ocular drug delivery. The most popular and convenient way is the conventional liquid ophthalmic formulation. Although ocular drops are well-accepted from patients, rapid precorneal elimination, dilution and nasolacrimal drainage of these drugs can cause low bioavailability and therapeutic response and also the frequent instillation of eye drops induces toxic side effects and cellular defacement; therefore, it may not be adequate for the patients (1).

To overcome some drawbacks of the eye drops including the quick removal of such drops and consequently the repeated instillation, several ophthalmic preparations have been suggested such as gels and ointments (viscous semi-solid forms).However, blurred vision (ointment, gel) and greasy feeling (ointment), as the disadvantages of these drugs, are not satisfactory to the patients (2).

To overcome the drawbacks mentioned before, hydrogel dosage forms can be used. Hydrogels are polymer networks that can modulate the controlled and sustained release of the drug. Moreover, good stability, biocompatibility with human body and resemblance to natural living tissue make *in situ* hydrogels more reliable for drug delivery. Application of biodegradable water-soluble polymers makes treatment more acceptable for patients. Currently, two groups of hydrogels are distinguished, namely preformed and *in situ* forming gels. Preformed hydrogels are the simple viscous solutions or hydrogel films, which gel outside of the eye and do not undergo any modification after administration. The blurred vision and lacrimation are the other drawbacks of these hydrogels (1). *In situ* forming polymeric formulations, which undergo sol-gel transition, are drug delivery systems that are in sol form before administration in the body, but once administered, undergo gelation *in situ*, to form a gel. The formation of gels depends on factors like temperature modulation, pH change, presence of ions and ultra violet irradiation, from which the drug gets released in a sustained and controlled manner. The *insitu* forming hydrogels containing the polymers with reversible sol-gel transition, are affected by different factors including the pH change, ion-activated systems, and temperature response. The pH change: with the presence of cellulose acetate phthalate (CAP) (3) and carbopol (4), variation of pH (ex: in formulated pH, the preparation is liquid and undergoes a rapid transition into viscous gel at the pH of tear fluid) can cause sol-gel transition. Ion-activated systems: when solutions instilled into the cul-de-sacarea, monomers of alginic acid (Glucuronicacid) or Gelrite^®^ (low-acetyl gellan gum) are contacted with tear fluid electrolytes, especially Na^+^ , Ca^2+^, and Mg^2+^cations and then the ionic hydrogels are finally formed. Temperature response: more than 20 years, many scientists are interested in working on the thermo-sensitive hydrogels and polymers. These hydrogels are liquid at preparation temperature (20-25 °C), but turn into a gel following an exposure to the body fluids as a result of increasing temperature. Different substances are triggered to temperature, the most useful copolymers are triblock copolymers of poly (ethylene oxide), poly (propylene oxide), poly (ethylene oxide) (PEO-PPO-PEO) (5,6), triblock copolymers of poly (ethylene glycol), poly (lactic acid), poly (ethylene glycol) (PEG-PLA-PEG) (7), and acrylic derivatives such as poly (N-isopropyl acrylamide) (PNIPAM) (6, 8). Poloxamer or pluronic series are the most useful copolymers, as mentioned before, with the patter of ABA, in which A is poly (ethylene oxide) (PEO) (30%) and B is also poly (propylene oxide) (PPO) (70%) (9, 10).

Carbomer is a poly-acrylic acid (PAA) polymer. When the pH is higher than the PKa (5.5), sol-gel conversion will take place. Carbomer is commercially named carbopol (11-13).

Loratadine is a second-generation histamine H1 receptor antagonist used in the treatment of allergic rhinitis and urticaria. It is indicated for the symptomatic relief of allergies (e.g., hay fever, urticarial) and to a limited extent, for asthma. For allergic rhinitis, loratadine is effective for both nasal and eye symptoms (e.g., sneezing, runny nose, itchy, or burning eyes). It is well-tolerated and 10 mg daily is effective for symptom relief (14).

## Experimental

*Materials*

Loratadine was prepared from Abidi pharmaceutical company (Tehran, Iran). Pluronic was obtained from Merck. Carbomer‌‌‌‌940 (Carbopol); Chitosan; and polyethylene glycol (PEG) were purchased from Merck (Germany). Cellulose acetate membrane 12kDa cut-off was prepared from TobaAzma (Tehran, Iran). All other materials were of the highest quality commercially available. 

*Animals*

Male adult Albino News eland rabbits (weighing 2.5- 3kg) were purchased from the laboratory animal center, Jundishapur University of Medical Sciences, Ahvaz, Iran. All animal experiments were carried out in accordance with the Ethical Committee of Ahvaz Jundishapur University of Medical Sciences (ref no. 826). The procedures were followed with regard to the standard international guidelines (15).

*Loratadine assay*

Determination of the amount of loratadine was carried out by UV spectrophotometry (BioWaveII, WPA) at λmax = 276 nm.

*Hydrogel preparation*

Hydrogel formulations were prepared using the full-factorial design. A calculated amount of carbopol (1.25%, 2.5% and 5%) was gradually added to some part of total water of formulation by using a stirrer at 60 °C for 4 h. Poloxamer (5-10%) was mixed with the remainder of the water formulation and was kept in 4 °C for at least 1h until a homogeneous mixture was obtained. At the next stage, the poloxamer solution was gradually added to the viscose solution of carbomer 940 under the magnetic stirring. Then drug (0.625%, 0.75%, 1.25%, 1.5% and 3%) and polyethylene glycol (PEG) (1%) and ethanol (1%) were added to the mixture of carbomer 940 and poloxamer under the continuous mechanical stirring (24 h) until the homogenous solution was obtained. Finally, 1ml of the 0.5% (w/v) acetate buffer solution was added to the resultant gel product until it was completely dissolved.

*Differential scanning calorimetry (DSC)*

To study the behavior of nanoparticles under thermal stress, differential scanning calorimetry (Mettler Toledo, Switzerland) was used. Interaction between ingredients and loratadine were studied through heating programs. Samples at first heated to 60 °C and kept at this temperature for 5 min to remove their thermal history. Then the temperature was reduced to 0 ͦ C (scan rate: 5 °C/ min). Samples kept at this temperature for 5 min and temperature was increased to 170 °C with the same rate. 

*In-vitro Drug release study*

Franz diffusion cells (area 3.4618 cm^2^) with a cellulose membrane were used to determine the release rate of loratadine from different hydrogel formulations. The cellulose (molecular weight G12 000) membrane was first hydrated in the distilled water solution at 25 °C for 24 h. The membrane was then clamped between the donor and receptor compartments of the cells. Diffusion cell was filled with 25 mL of hydrochloric acid 0.1 M. The receptor medium was constantly stirred by the externally driven magnetic beads at 300 rpm throughout the experiment. Loratadine hydrogels (3g) were accurately weighed and placed in the donor compartment. At predetermined time intervals (0.5, 1, 2, 3, 4, 5, 6, 7, 8, 9, 10, 11, 12, and 24 h); 2mL sample was removed from receptor for the spectrophotometric determination and replaced immediately with an equal volume of fresh receptor solution. The samples were analyzed by a UV-visible spectrophotometer (BioWaveII, WPA) at 276 nm. The results were plotted as cumulative amount of drug released versus time (16).

**Table 1:** Compositions of Selected Hydrogels of Loratadine, Swellingand Porosity Ratios and 24- h. Drug Released Percent (mean ± SD, n = 3).

**code**	**Factorial state**	**Pluronic** **(%)**	**Carbomer 940** **(%)**	**Drug** **(%)**	**Ethanol+** **PEG (1:1)** **(%)**	**Water** **(%)**	**Swelling Ratio**	**Porosity** **Ratio**	**24 h Drug released** **(%)**
F1	+ + +	10	2.5	1.25	2	84.25	41.85±4.1	1.38±0.14	56.56±1.2
F2	+ + -	10	2.5	2.5	2	83	43.16±3.2	1.32±0.01	48.85±0.9
F3	+ - -	10	5	3	2	80	37.58±1.3	1.48±0.33	41.03±1.1
F4	+ - +	10	5	1.5	2	81.5	35.1±1.7	1.35±0.11	58.87±1.3
F5	- + -	5	1.25	1.25	2	90.5	37.15±3.8	0.15±0.02	78.41±1.6
F6	- + +	5	1.25	0.625	2	91.125	44.92±3.9	0.078±0.01	17.36±0.8
F7	- - +	5	2.5	0.75	2	90.75	68.66±3.5	0.033±0.04	98.9±1.2
F8	- - -	5	2.5	1.5	2	89	33.14±3.6	0.64±0.1	76.85±1.4

**Figure 1:**
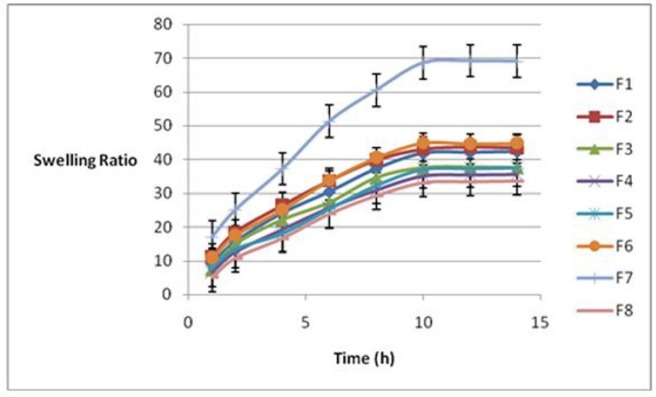
Swelling ratio of hydrogels as a function of time at 37 °C

**Figure 2:**
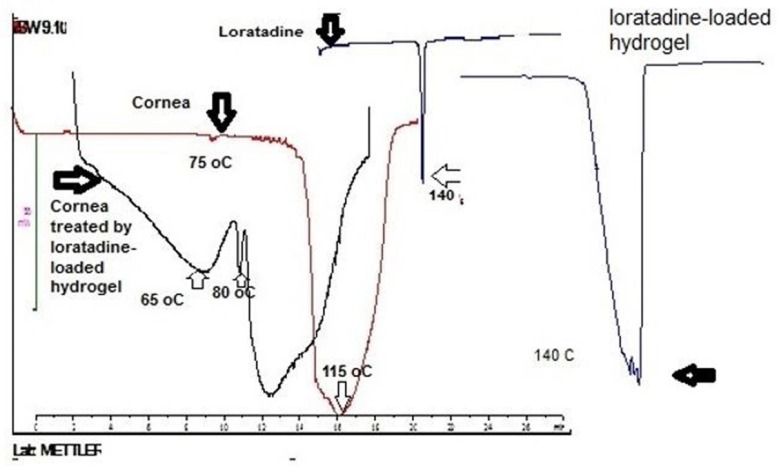
DSC thermograms of loratadine, cornea and cornea treated with loratadine loaded – hydrogel.

**Figure 3:**
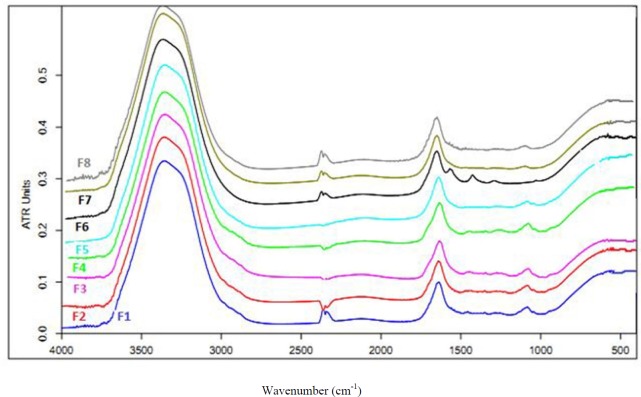
FT-IR spectra of the prepared hydrogel formulations

**Figure 4:**
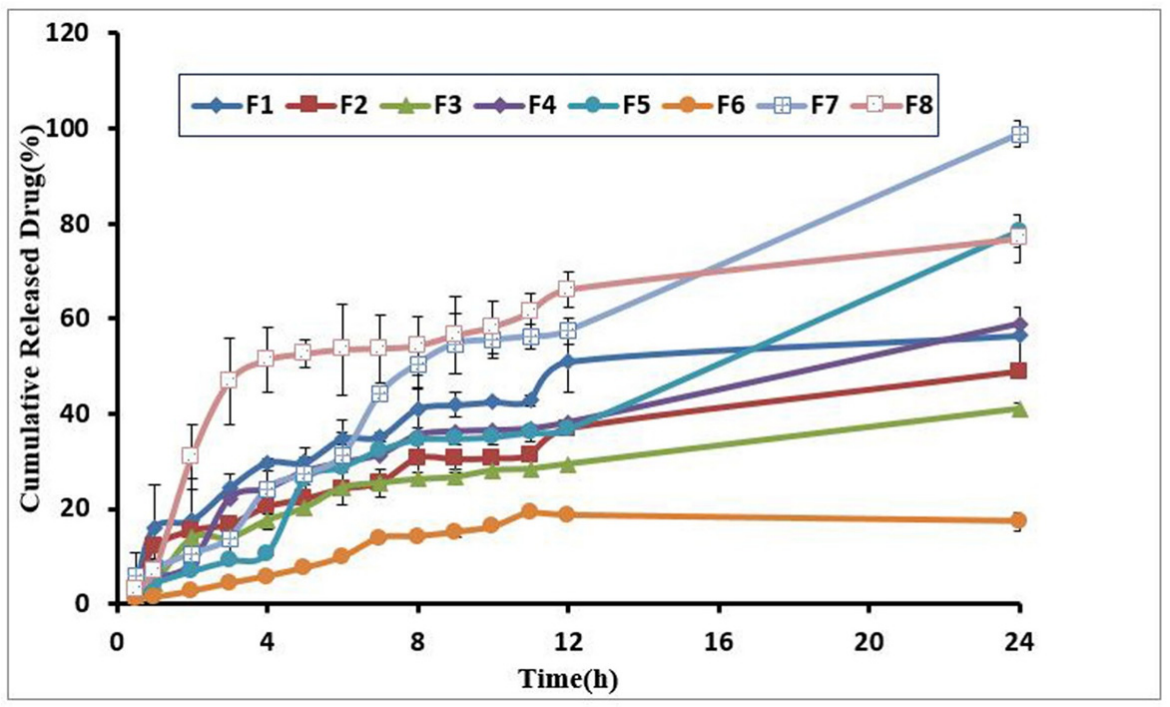
Drug release profiles through hydrogel formulations

**Figure 5:**
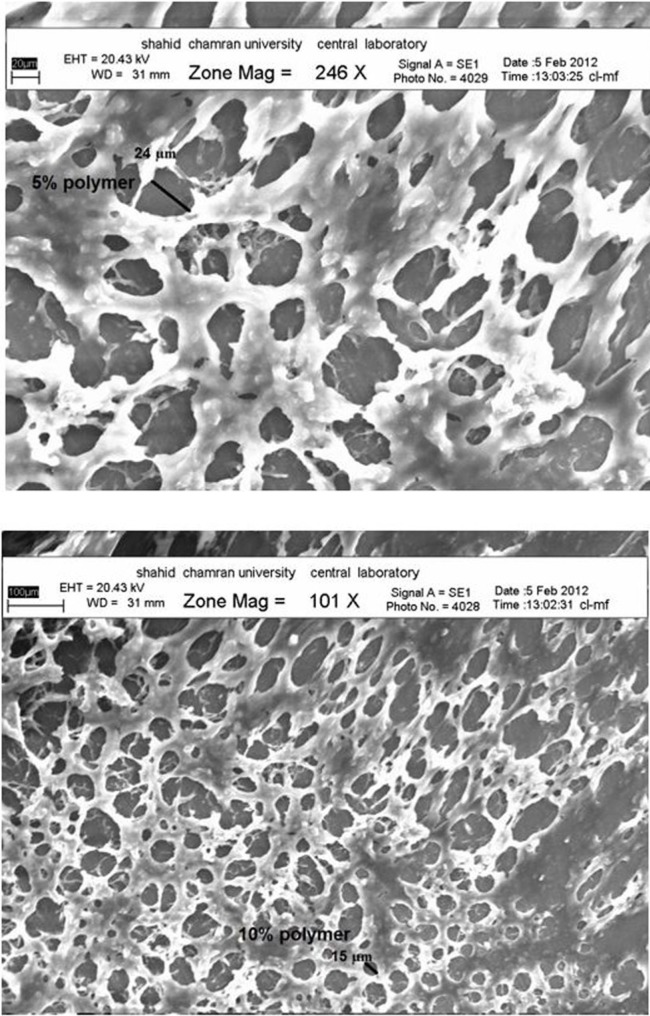
SEM images of hydrogel F7

**Figure 6:**
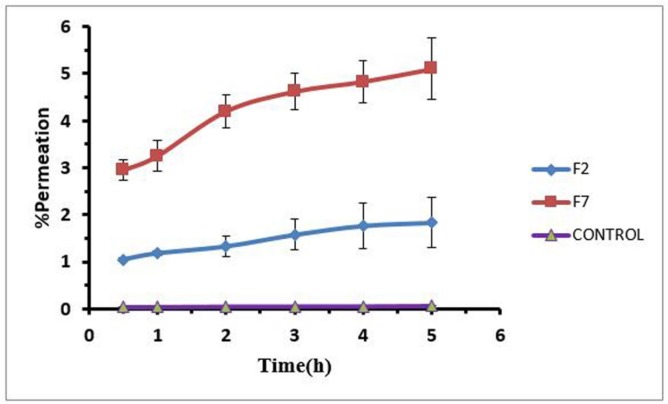
*In-vitro* Permeation profiles of Loratadine hydrogel (F2, F7) through rabbit cornea after 5 h.

*The Ex vivo cornea permeation studies*

The eye cornea with sclera ring was separated from a newly sacrificed Albino rabbit. The subcutaneous tissue was completely removed without causing any injury using the scissors or a scalpel. The removed tissue was kept in a glutathione buffer. The *in-vitro* cornea permeation studies were carried out using vertical glass diffusion cells fabricated in house with an effective diffusion area of approximately 1.53 cm^2^. The volume of the receptor section was 7-ml.The cornea was mounted between the donor and receptor compartments of the cell in this manner that sclera ring clamped between two chambers and cornea facing the receptor without any damage due to diffusion cell apparatus. The equilibrated diffusion cell was maintained at 37 °C ± 0.1 for 1h. The receptor medium was constantly stirred using the externally driven magnetic beads at 300 rpm throughout the experiment. Simulated tear fluid (STF) has received phase stirred permanently at 37 °C and 300 rpm. At each interval times (0.5, 1, 2, 3, 4, 5 h), a 2 mL sample was withdrawn from the medium, and immediately replaced with an equivalent volume of fresh STF. The permeated amount of loratadine was determined using the UV spectroscopy method at 276 nm.

*Swelling characterization*

To determine the dynamic swelling behavior, Hydrogels weighed and soaked in excess of simulated tear fluid (STF) at 37 °C to reach the swelling equilibrium. The STF was comprised of NaCl (0.67 g), NaHCO3 (0.2g), CaCl_2 _(0.008 g), and deionized water (100 g). The excess surface water in the swollen gel was removed by blotting and then the swollen gel was weighed again. Swelling percent (Q) was calculated from the below equation:

Q = (W_s_− W_d_)/W_d_

Where W_s _and W_d _are the weight of swollen hydrogel and dried hydrogel, respectively. The water uptake was plotted against time to show swelling reached the equilibrium. 

*Porosity measurement*

The porosity of hydrogels was determined using the solvent replacement method. Porosity can be measured using a displacement liquid that is not a solvent of the polymer, for example ethanol, and is able to penetrating into pore size without any size shrinkage or swelling (17). Therefore, based on preformulation study, ethanol was selected as displacement liquid in this study. Dried hydrogels were immersed overnight in the absolute ethanol and weighed after the excess ethanol on the surface was blotted. The porosity was calculated from the following equation:

Porosity = (M_2_ – M_1_) / ρV

Where M1 and M_2 _are the mass of the hydrogel before and after an immersion in absolute ethanol, respectively; ρ is the density of absolute ethanol and V is the volume of the hydrogel.

*FT-IR studies*

Nature of the interacting forces can be changed during gelation process. This can be observed with a Fourier transform infrared spectroscopy. Hydrogel samples were dried with atmospheric condition for 24 h. The samples were scanned in the range 4000 to 400 cm^-1^ using an FT-IR facility (Uker, Vertex70, and Germany).

*Experimental design study*

Several parameters have an effect on final hydrogel properties and permeation through rabbit cornea. The full-factorial design was used concerning three variables at two levels for formulations (Table 1). Major variables in determination of hydrogel’s properties include Pluronic percentage, Pluronic/carbomer ratio (p/c), and drug/ (Pluronic + carbomer) ratio (d/p+c). Eight different formulations with low and high values of pluronic percentage (5% and 10%), Pluronic/carbomer ratio (2:1, 4, 1), and drug/ (Pluronic + carbomer) ratio (1:5, 1:10) were used to prepare the hydrogel formulations. Dependent variables include the porosity, swelling, drug release and permeation parameters through rabbit cornea. The effect of independent variables was evaluated versus dependent variables.

*Morphological characterizatio* *n*

The SEM specimens were mounted onto Al-stub, dried by liquid N_2_, and then counted by gold/palladium using an accelerating voltage of 20 KV (18). 

*Data analysis and statistics*

All the experiments were repeated three times and data were expressed as the mean values. Statistical data were analyzed using the one-way analysis of variance (ANOVA) and *P *< 0.05 was considered to be significant with a 95% confidence interval. To figure out the relationship between dependent and independent variables, the simultaneous multi-regression test was used.

## Results and Discussion

*Swelling properties of hydrogels*

Figure 1 indicates swelling ratios plotted against time for all formulations. As it is shown, in this figure swelling reached the equilibrium in 10 h. Therefore, swelling ratio after 10 h was selected as a sign of hydrogel swelling property. Table 1 illustrates the swelling ratios for various formulations. The results showed a significant correlation between the swelling and porosity rates of the hydrogels with the pluronic percentage (*P *= 0.03) and p/c ratio (*P *= 0.037). Formulation 7 (F7) and formulation 3 (F3) have the highest and the lowest swelling ratios, respectively. The equation 1 indicated that the pluronic percent (%P) had the most significant effect on swelling of the formulations (*P *= 0.03). There was an inverse relationship between the swelling ratio and the percentage of Pluronic, that is, the swelling ratio decreased with an increase in the Pluronic.

Equation1: Swelling after 10 h = 33.1-5.9 (%P) -32.6(P/C) + 52.9 d/p+c

It can be easily observed that there is an increase in the swelling ratio when %p equals to 5%.

*Porosity of the hydrogels*

Results of porosity (Table 1) showed that F3 (porosity = 1.48) had the maximum and F7 (porosity = 0.32) the minimum porosity. To understand the effect of independent variables on porosity of formulations, results were evaluated using the regression and factorial analysis (equation 2).

Equation 2: Porosity after 12 h = - 0.441 + 0.232 %p - 0.0712 p/c - 1.87 d/p + c

A significant relation was observed regarding %p (*P *= 0.03) and p/c ratio (*P *= 0.04). Porosity was increased with the increase of %p and decrease of p/c ratio. It seems that the porosity controlled by the Pluronic percentage.

*Differential scanning calorimetery*

In this research, DSC was performed to study the interaction between loratadine loaded in hydrogel and cornea. Figure 2 illustrates thermograms of loratadine, cornea and cornea treated with loratadine loaded- hydrogel. Loratadine indicated an endotherm at 140 °C corresponding to melting point that is in accordance with literature (19). However, the endothermic disappeared in cornea treated with loratadine-loaded in hydrogel. It means the loratadine interacted with cornea and dispersed with molecular dimension over that. On the other hand, cornea demonstrated two endothermic transitions around 75 and 115 °C that after treatment with loratadine-loaded hydrogel shifted to lower temperature. These results suggest loratadine-loaded hydrogel interacted with cornea that is in accordance with mucoadhesive property of polymer.

*Evaluation of hydrogel structure by the FT-IR technique*

To assess the vibration bands of polymers and nature of the interacting forces during gelation process, all the prepared hydrogel formulations were scanned in the range of 400-4000 cm^-1 ^with a speed of 2 cm/sec with the resolution of 4 cm^-1^. Figure 3 shows the FT-IR spectra of the hydrogels. Three main vibrational bands were found in the spectra of the hydrogels. A band at 3300-3500 cm^-1^in the spectra is attributed to the hydroxyl (OH) stretching vibration of the carbomer chains. Another band is seen at 1620- 1640 cm^-1^ representing the carbonyl (C = O) stretching in the carbomer. Third band was found in 1000-1100 cm^-1^ that is attributed to ether band (C-O-C) in Pluronic that is disappeared in the presence of loratadine. Results of this study revealed an interaction between loratadine and Pluronic that is a main reason for drug-loading. Moreover, a direct and significant correlation was found between the percentage of Pluronic and the wave numbers of hydroxyl group in the carbomer chain. Therefore, the hydroxyl groups are slightly shifted to higher frequencies because of an interaction between Pluronic and carbomer; that is an obvious reason for sol-geltransition. This correlation has been shown for the percentage of Pluronic and wave numbers of carboxylic group in carbomer. Therefore, Pluronic interacts with hydroxyl and carboxylic groups in carbomer, which is a main reason for the hydrogel network formation and interacts with loratadine, which is the main reason for drug-loading in polymer.

*Drug release study*

To evaluate the influence of the independent variables on drug release of formulations, once we studied the amounts of drug release as a response and by plotting a drug release curve against time, a well-characterized drug release description was obtained. Furthermore, to determine the effect of independent variables on drug release and recognize the difference between the formulations regarding release profile, the percentages of a drug released after 2 h (D_2_) and 24 h (D_24_) were measured. D_2_ concerns with rapid release of the component whereas D_24_ indicates the slow release rate. Results revealed that the percentage of Pluronic had an indirect significant relation with D_2_ (*P *= 0.02), whereas the other three variables had no significant effect. Elevation in Pluronic was a sign of a decrease in drug release. The findings of this study confirmed the FT-IR spectrum analysis that indicated an interaction between loratadine and Pluronic. All the formulations showed the sustained release profiles that only an approximate average of 17-98% of the loaded drug was released from the hydrogels after 24 h. There was an indirect significant correlation between the percentages of D_24_ and D_2_ with the amount of Pluronic polymer. Drug release profile demonstrated the low-burst effect and good sustained release property. Table 1 indicated the drug release percent after 24 h from the hydrogels and Figure 4 drug release profiles from the hydrogels.

*Morphological examination*

Following picture (Figure 5) shows the morphology of the porous structure of hydrogels with 5 and 10% polymer. It can be seen that the inner pores of hydrogels are interconnected with the irregular shapes. The scanning electron microscopy (SEM) images showed that the pore size was decreased slightly with increasing of the polymer concentrations in the hydrogel may due to an increase in the number of hydrophobic moieties in the hydrogel. The introduction of physical cross-linking through the hydrophobic interaction of the polypropylene oxide (PPO) segments in the Pluronic and carbomer chain is believed to be responsible for producing the three-dimensional network structures.

*Drug permeation through rabbit cornea*

To evaluate the effect of formulations on loratadine permeation through rabbit cornea, F2 and F7 hydrogels were selected because the F2 and F7 have higher and lower percent of polymer and drug respectively. The amount of permeated drug was measured during 5 h for both formulations against aqueous solution of drug as control with viscosity around 25-30 cps that was comparable with viscosity of hydrogels. Drug permeated by both formulations significantly increased compared to the control group. Drug permeation percentages for the F2 and F7 formulations were 15 and 70 folds more than that of the control. It seems that drug release from hydrogels did not limit drug permeation through the cornea. The difference between the two formulations is only in the percentage of polymer. Higher level of Pluronic in F2 and higher level of carbomer in F7 made different drug release and permeation in this manner so that F2 provided the lower drug release and permeation and F7 provided the higher drug release and permeation. Figure 6 shows *in-vitro* permeation profiles of loratadine hydrogel (F2, F7) through rabbit cornea.

The selected hydrogels increased the permeability of loratadine, as lipophilic drug, through rabbit cornea. It seems that the effect of hydrogel formulations was due to the mucoadhesive property of hydrogel that increases the drug retention time. This finding suggests that the formulation characterization is the main factor for the determination of drug permeability through rabbit cornea, especially the amount of carbomer with mucoadhesive property.The administration of mucoadhesive factors (carbomer) can lead to longer resistance time of the dosage form in the eye because the drug would turn into gel structures at the body temperature. Therefore, a system consisting of both Pluronic and carbomer will be of great interest because it utilizes both thermogelling and mucoadhesive properties, which can be the perfect characters for an ocular drug delivery. The appearance of hydrogels is clear and they may not blur the vision but to prove it *in-vivo* experiment is needed. 

## Conclusions

In conclusion, selected hydrogels increased loratadine as lipophilic drug permeation through rabbit cornea. It seems that the effect of hydrogel formulations was due to mucoadhesive property of hydrogel that increases the drug retention time. Drug partitioning into the cornea is the first step of penetration and it seems that hydrogel formulations increased it. FT-IR spectra indicated an interaction between pluronic and carbomer that makes hydrogel network and also it indicated an interaction between pluronic and loratadine that is the main reason of drug loading. Drug loading efficiency for lipophilic compounds like loratadine in the hydrogel with hydrophilic nature was suitable. Drug release profile demonstrated the low-burst effect and good sustained release property. This finding suggests that the formulation characterization is the main factor for the determination of drug permeability through rabbit cornea, especially amount of carbomer with mucoadhesive property. 

## References

[1] Wei G, Xu H, Ding PT, Li SM, Zheng JM (2002). Thermosetting gels with modulated gelation temperature for ophthalmic use: the rheological and gamma scintigraphic studies. J. Control Release.

[2] Nanjawade BK, Manvi F, Manjappa A (2007). RETRACTED: In situ-forming hydrogels for sustained ophthalmic drug delivery. J. Control. Release.

[3] Yin L, Fei L, Tang C, Yin C (2007). Synthesis, characterization, mechanical properties and biocompatibility of interpenetrating polymer network–super-porous hydrogel containing sodium alginate. Polym. Int.

[4] Srividya B, Gardozza RM, Amin PD (2001). Sustained ophthalmic delivery of ofloxacin form a pH triggered situ gelling system. J. Control. Release.

[5] Alexandridis P, Hatton TA (1995). Poly(ethylene oxide) poly(propylene oxide) poly(ethylene oxide) block copolymer surfactants in aqueous solutions and at interfaces: thermodynamics, structure, dynamics and modeling. Colloids and Surfaces A.

[6] Haffman AS (2002). Hydrogels for biomedical applications. Adv. Drug Deli. Rev.

[7] Jeong B, Bae Y, Lee D, Kim S (1997). Biodegradable block copolymers as injectable. Drug Deliv. Systems Nature.

[8] Peppas N, Bures P, Leobandung W, Ichikawa H (2000). Hydrogels in pharmaceutical formulations. Eur J. Pharm. Biopharm.

[9] Ludwig A (2005). The use of mucoadhesive polymers in ocular drug delivery. Adv. Drug. Deliv. Rev.

[10] Ruel-Gariépy E, Leroux J-C (2004). In situ-forming hydrogels—review of temperature-sensitive systems. Eur. J. Pharm. Biopharm.

[11] Zoppi A, Linck YG, Monti GA, Genovese DB, Jimenez Kairuz ÁF, Manzo RH (2012). Studies of pilocarpine: carbomer intermolecular interactions. Int. J. Pharm. Sci.

[12] Hosseinzadeh H, Atyabi F, Dinarvand R, Ostad SN (2012). Chitosan–Pluronic nanoparticles as oral delivery of anticancer gemcitabine: preparation and in-vitro study. Int. J. Nanomedicine.

[13] Jeong B, Gutowska A (2002). Lessons from nature: stimuli-responsive polymers and their biomedical applications. Trends Biotechnol.

[14] Sharif Makhmal zadeh B, Dahanzadeh S, Rahim F (2011). Preparation and evaluation of the self emulsifying drug delivery system containing loratadine. Int. J. Advances Pharm. Sci.

[15] Office of Laboratory Animal Welfare (2002). National Institutes of Health, Public Health Service Policy on Human Care and Use of laboratory animals. Bethesda.

[16] Salimi A, Moghimipour E, Rahmani F (2015). Effects of the Various Solvents on the In-vitro Permeability of Indomethacin through Whole Abdominal Rat Skin. Annu. Res. Rev. Biol.

[17] Shi G, Cai Q, Wang C, Lu N, Wang S, Bei J (2002). Fabrication and biocompatibility of cell scaffolds of poly (L-lactic acid) and poly (L-lactic- co –glycolic acid). Poly. Adv. Technol.

[18] Moghimipour E, Salimi A, Karami M, Isazadeh S (2013). Preparation and Characterization of Dexamethasone Microemulsion Based on Pseudoternary Phase Diagram. Jundishapur J. Nat. Pharm. Prod.

[19] Ramos LA, Cavalheiro ETG (2007). Thermal behavior of loratadine. J. Thermal. Anal. Calorimet.

